# Machine learning-based prediction of in-hospital mortality for critically ill patients with sepsis-associated acute kidney injury

**DOI:** 10.1080/0886022X.2024.2316267

**Published:** 2024-02-18

**Authors:** Tianyun Gao, Zhiqiang Nong, Yuzhen Luo, Manqiu Mo, Zhaoyan Chen, Zhenhua Yang, Ling Pan

**Affiliations:** a Department of Nephrology, The First Affiliated Hospital of Guangxi Medical University, Nanning City, PR China; b Department of Critical Care Medicine, The First Affiliated Hospital of Guangxi Medical University, Nanning City, PR China

**Keywords:** Sepsis, acute kidney injury, prediction model of prognosis, machine learning algorithms

## Abstract

**Objectives:**

This study aims to develop and validate a prediction model in-hospital mortality in critically ill patients with sepsis-associated acute kidney injury (SA-AKI) based on machine learning algorithms.

**Methods:**

Patients who met the criteria for inclusion were identified in the Medical Information Mart for Intensive Care-IV (MIMIC-IV) database and divided according to the validation (*n* = 2440) and development (*n* = 9756, 80%) queues. Ensemble stepwise feature selection method was used to screen for effective features. The prediction models of short-term mortality were developed by seven machine learning algorithms. Ten-fold cross-validation was used to verify the performance of the algorithm in the development queue. The area under the receiver operating characteristic curve (ROC-AUC) was used to evaluate the differentiation accuracy and performance of the prediction model in the validation queue. The best-performing model was interpreted by Shapley additive explanations (SHAP).

**Results:**

A total of 12,196 patients were enrolled in this study. Eleven variables were finally chosen to develop the prediction model. The AUC of the random forest (RF) model was the highest value both in the Ten-fold cross-validation and evaluation (AUC: 0.798, 95% CI: 0.774–0.821). According to the SHAP plots, old age, low Glasgow Coma Scale (GCS) score, high AKI stage, reduced urine output, high Simplified Acute Physiology Score (SAPS II), high respiratory rate, low temperature, low absolute lymphocyte count, high creatinine level, dysnatremia, and low body mass index (BMI) increased the risk of poor prognosis.

**Conclusions:**

The RF model developed in this study is a good predictor of in-hospital mortality for patients with SA-AKI in the intensive care unit (ICU), which may have potential applications in mortality prediction.

## Introduction

Sepsis-associated acute kidney injury (SA-AKI) is a common and serious complication in critically ill patients. A European multicenter study showed that 51% of patients with sepsis and the intensive care unit (ICU) were complicated with AKI, and the death rate of SA-AKI patients was 41% [1]. Septic AKI patients in the ICU were more likely to have a greater burden of illness, higher mortality, and requirements for dialysis than patients with nonseptic AKI [2,3]. Early identification of high-risk individuals and effective intervention are helpful for improving prognosis and survival in patients with SA-AKI [4,5]. The pathogenesis of SA-AKI is complicated and not completely clear, and it is difficult to find a single sensitive biomarker [6]. A prediction model that involves multiple related risk factors may be a better choice to solve this problem.

Some previous studies have developed prediction models of mortality or poor prognosis for patients with SA-AKI based on the Medical Information Mart for Intensive Care (MIMIC)-III dataset or the ICU data of their hospital. These studies are generally based on general severity scores, combined with population data, comorbidities and infection indicators, renal function, and other relevant indicators, and have shown some effectiveness in predicting the prognosis of SA-AKI [6–8].^.^ The MIMIC-IV database is the latest MIMIC database, compared to MIMIC-II and MIMIC-III, and contains information regarding a patient’s entire hospital stay. MIMIC-IV contains clinical data from more than 60,000 patients who were hospitalized in the ICU at Beth Israel Deaconess Medical Center between 2008 and 2019. Few studies have focused on SA-AKI data from MIMIC-IV until now.

But selecting appropriate and significant predictors of the prediction model is a major challenge for all kinds of indices. The majority of prior studies concentrated on full factorial models, lacked efficient feature screening methods, and models incorporated more factors. In big medical data, machine learning methods can handle multicollinearity of independent variables with more convenience, be used to increase the prediction discrimination, accuracy, and stability of prognosis prediction models compared with traditional regression analysis [9,10]. Model construction techniques of machine learning methods, which include random forest (RF), extreme gradient boosting (XGBoost) and other methods, have been widely used in the medical field [11,12]. Integrating feature ranking and screening predictors step by step and obtaining a subset of valid features were also helpful for improving the discrimination and accuracy of a prediction model [13]. The new variable screening methods in combination with multiple machine learning techniques may further increase modeling effectiveness. The large amount of detailed and continuously updated clinical data, combined with data-driven machine learning techniques, enables the efficient processing of complex fitting relationships in big data and the development of new mortality prediction tools.

Using MIMIC-IV data, this study aims to identify risk factors and develop a prediction model of in-hospital mortality among patients with SA-AKI in the ICU using multiple machine learning algorithms. It is beneficial for predicting short-term mortality in high-risk patients with SA-AKI in the ICU.

## Materials and methods

### Data source

The information was obtained from the sizable, publicly available MIMIC-IV (version 1.0) critical care database, which includes vital signs, medications, laboratory test results, comorbid diagnoses, imaging reports, survival data, and other health-related data on patients admitted to the ICU at Beth Israel Deaconess Medical Center from 2008 to 2019 [14]. We were given access to the database through the protection of human research participants assessment (Certificate No. 42064390). This database may be used by any researcher who complies with the data user requirements, according to approval from the Institutional Review Boards of Massachusetts Institute of Technology. Data extraction was carried out using Structured Query Language (SQL). The primary outcome of the prediction model was in-hospital mortality. The development queues and the validation queues were split with an 8:2 ratio of the study population.

### Study population

If the following criteria were met, patient records were extracted from the MIMIC IV database for this study: (1) age ≥ 18 years, (2) met the Kidney Disease: Improving Global Outcomes (KDIGO) diagnostic criteria for AKI, and (3) met the 3rd edition of internationally accepted diagnostic criteria for the definition of sepsis (Sepsis-3). The following exclusion criteria were used: (1) patients with chronic kidney disease (CKD) stage 5 (eGFR < 15 or those who received long-term renal replacement therapy), (2) follow-up time less than 48 h (for patients with repeated hospitalizations, only information from the first hospitalization was included). The follow-up period solely covered the current hospitalization, ending with the current discharge (Supplemental Figure 1).

### Definitions

The diagnosis of sepsis was in accordance with Sepsis-3.0, with specific criteria of sequential organ failure assessment (SOFA) score ≥2 and infection or suspected infection [15]. The diagnosis and staging of AKI was in accordance with the 2012 KDIGO guidelines: an increase in serum creatinine (SCr) level of 0.3 mg/dL within 48 h or an increase to 1.5 times the baseline Scr level within the past 7 d [16].

### Data extraction

Patient information was extracted in MIMIC-IV database using PostgreSQL 13 software. Within 24 h of the patient’s admission, basic data, vital signs, laboratory test indicators, condition score scales, and survival data were gathered. To obtain diagnostic data, comorbidities were identified using the International Classification of Diseases diagnosis codes. Variables with more than 25% missing data were excluded to lessen the bias brought on by missing data. Consequently, the number of prepared features is 51. When the percentage of missing values was less than 25%, the miceforest package of Python software was used to fill in the missing values of the variables using multiple imputation [17].

### Ensemble stepwise feature ranking and selection

We extracted demographic information, routine vital signs, laboratory values, scores, comorbidities, and medications as features from patients’ admission information and charted data which is clinically readily available. As a result of having additional indirectly connected hyperparameters, individual predictors are prone to overfitting by producing an excessive number of features. To facilitate clinical applications and reduce the influence of noise and irrelevant variables, we used ensemble stepwise feature ranking and selection to perform a stepwise integration method for feature ranking and selection, selecting some valid features from the whole feature set to form a model feature set.

RFs are frequently employed for feature screening prior to modeling. We first ranked the importance of features using RF, which calculates the importance of variables by calculating the average information gain (Gini index)[18]. Begin to suppose we have M predictors. We then compute the ensemble output using bagging. The complete dataset was divided into M subsets, with one fold serving as a validation set and the others as training sets. As a result, the training/validation set and the development set are split into M halves. On the basis of them, we can then construct M feature ranker. Using cross-validation, we will divide the full development set into segments and then create a subset from those segments. To rank features, we will resort to feature significance. Then the ensemble feature ranking can be obtained.

To create final features set, we first choose the best features for each iteration based on the feature ranker. We make iterative addition of one new feature to the already selected feature set, and in the feature selection process that involved assembling M predictors based on split. We then analyze the mortality predictor on the validation set after training it. To speed up the computation, the logistic regression (LR) classifier was chosen as the predictor. In the end, we use 10-fold cross-validation to calculate the average performance of predictors with top features. We compute the average performance of predictors using receiver operating characteristic (ROC) curves, and the area under the ROC curves (ROC-AUC) as the measurements. Finally, output the number of features who has the best performance.

### Model development and evaluation

The development queues was used to confirm the algorithm performance by ten-fold cross-validation, the average of ROC-AUC in ten-fold cross-validation was calculated, and the ROC curve was plotted. The test set was used to confirm the discrimination and calibration of the model, whereas the development queues was used to build the model and select features. The features were filtered based on the stepwise integration of feature selection and feature ranking. The data were normalized and fed into seven machine learning algorithms: K-nearest neighbors (KNN) [19], extreme gradient boosting [20], naive Bayesian (NB) [21], decision tree [22], support vector machine (SVM, linear/rbf) [23], RF [24], and LR [25]. The ROC-AUC values, accuracy, precision, and F1 score (2* ((precision*recall)/(precision + recall))) were compared to evaluate the best prediction models and perform internal validation [26].^.^ The Delong test was used for AUC comparison.

Hyperparameter:

KNN: KNeighborsClassifier(n_neighbors = 3);

NB: GaussianNB(priors = None);

DecisionTree:DecisionTreeClassifier (*,criterion="gini", splitter="best", max_depth = None, min_samples_split = 2,min_samples_leaf = 1,min_weight_fraction_leaf = 0.0,max_features = None, random_state = None, max_leaf_nodes =None, min_impurity_decrease = 0.0, class_weight = None, ccp_alpha = 0.0);

SVM, linear/rbf: svm.SVC(kernel=’linear’, probability = True)/svm.SVC(kernel=’rbf’, probability = True);

Random Forest: RandomForestClassifier(n_estimators = 100,random_state = 0);

Logistic Regression: LogisticRegression(penalty="l2", *, dual = False, tol = 1e-4, C = 1.0, fit_intercept = True, intercept_scaling = 1, class_weight = None, random_state = None, solver="lbfgs", max_iter = 100, multi_class="auto", verbose = 0, warm_start = False, n_jobs = None, l1_ratio =None);

### Statistical analysis

Statistical analysis, modeling, and validation were implemented using Python version 3.8 software and module packages [27]. For normally distributed variables, the continuous variables were expressed as the mean ± standard deviation, and nonnormally distributed variables were expressed as the median (interquartile range). Categorical variables were displayed as percentages. In univariate analyses, categorical variables were compared using Pearson’s chi-squared or Fisher’s exact tests, and continuous variables were compared using Student’s t tests or the Kruskal–Wallis test as appropriate. *p* Values <0.05 were considered statistically significant. The area under the ROC curve (AUC), accuracy, precision and F1 score were used in the internal validation to compare the performance of the models constructed by the seven machine learning algorithms, and the model with the best performance was used as the final prediction model. Shapley additive explanations (SHAP) were used to explain the results of the best prediction model [28]. When the SHAP value of the variable in the sample is > 0, the variable has a positive effect on the prediction of the outcome at this time. The SHAP summary plot and SHAP dependence plot of the final prediction model were plotted to determine how each variable affected the prognosis of SA-AKI patients during hospitalization and how the positive and negative effects of the variables on outcome prediction varied with their values. The SHAP force plot for patients was plotted to demonstrate how the model personalizes the prediction of each patient’s condition and guides clinical decision-making.

## Results

### Patient characteristics

A total of 12,196 patients were included in the study. The mean age of these patients was 67.0 ± 16.1 years, of whom 6995 (57.4%) were men, with a male to female ratio of 5.7:4.3 and a mean length of stay of 15 d. The in-hospital mortality rate was 19.3% (2352/12,196). The baseline demographic and clinical characteristics of SA-AKI patients who died or survived during hospitalization are shown in Table 1. In the admission score, the Simplified Acute Physiology Score (SAPS II) and SOFA score were higher and the Glasgow Coma Scale (GCS) score was lower in the nonsurviving group than in the surviving group. The length of ICU stay was longer in the nonsurvivor group, but the length of hospitalization was shorter.

**Table 1.** Characteristics of the patients with sepsis associated AKI (SA-AKI).

Parameters	Total(*n* = 12,196)	Survival group(*n* = 9944)	Non-survival group(*n* = 2352)	*p* Value
Age (y)	67.0 ± 16.1	66.4 ± 16.2	69.5 ± 15.6	<0.001
Gender (male, %)	6995 (57.4)	5683 (57.7)	1312 (55.8)	0.090
BMI(kg/m2)	29.6 ± 7.8	29.8 ± 7.8	28.7 ± 7.8	<0.001
Baseline creatinine* (mg/dL)	1.0 ± 0.8	1.0 ± 0.7	1.1 ± 0.8	<0.001
Systolic blood pressure (mmHg)	115.4 ± 15.1	115.8 ± 14.9	113.6 ± 15.8	<0.001
Diastolic blood pressure (mmHg)	61.3 ± 10.4	61.3 ± 10.3	61.0 ± 10.8	0.132
Mean arterial Pressure (mmHg)	76.7 ± 10.1	76.9 ± 10.0	75.9 ± 10.6	<0.001
Respiratory rate (times per minute)	19.9 ± 4.1	19.6 ± 4.0	21.2 ± 4.4	<0.001
Temperature (°C)	36.9 ± 0.7	36.9 ± 0.6	36.7 ± 0.8	<0.001
SpO2 (%)	97.1 (2.2)	97.1 (2.0)	96.7 (2.7)	<0.001
AKI stage, n (%)				<0.001
Stage 1	2200 (18.0)	1991 (20.2)	209 (8.9)	
Stage 2	5877 (48.2)	5150 (52.3)	727 (30.9)	
Stage 3	4119 (33.8)	2703 (27.5)	1416 (60.2)	
Urine output(mL/d)	1674.5 ± 1205.7	1751.8 ± 1201.3	1351.0 ± 1170.0	<0.001
Creatinine(mg/dL)	2.0 ± 1.7	1.9 ± 1.7	2.6 ± 1.9	<0.001
ARDS, *n* (%)	5687 (46.6)	4471 (45.4)	1216 (51.7)	<0.001
Myocardial infarction, *n* (%)	2308 (18.9)	1822 (18.5)	486 (20.7)	0.018
Congestive Heart failure, *n* (%)	4010 (32.9)	3155 (32.0)	855 (36.4)	<0.001
Cerebrovascular disease, *n* (%)	2101 (17.2)	1591 (16.2)	510 (21.7)	<0.001
Chronic pulmonary disease, *n* (%)	3398 (27.9)	2723 (27.7)	675 (28.7)	0.326
Diabetes, *n* (%)	3669 (30.1)	2995 (30.4)	674 (28.7)	0.098
Renal disease, *n* (%)	2394 (19.6)	1858 (18.9)	536 (22.8)	<0.001
Malignant cancer, *n* (%)	1611 (13.2)	1146 (11.6)	465 (19.8)	<0.001
Hypertension, *n* (%)	5450 (44.7)	4522 (45.9)	928 (39.5)	<0.001
Charlson comorbidity index	5.9 ± 2.9)	5.7 ± 2.8)	6.9 ± 3.0)	<0.001
Cardiac Surgery, n (%)	2147 (17.6)	2053 (20.9)	94 (4.0)	<0.001
Mechanical Ventilation, n (%)	11764 (96.5)	9463 (96.1)	2301 (97.8)	<0.001
Dialysis, n (%)	1147 (9.4)	653 (6.6)	494 (21.0)	<0.001
Vasopressin, *n* (%)	1873 (15.4)	1046 (10.6)	827 (35.2)	<0.001
Dopamine, *n* (%)	651 (5.3)	426 (4.3)	225 (9.6)	<0.001
Epinephrine, *n* (%)	1083 (8.9)	828 (8.4)	255 (10.8)	<0.001
Norepinephrine, *n* (%)	4831 (39.6)	3321 (33.7)	1510 (64.2)	<0.001
Antifungal medications, *n* (%)	3605 (29.6)	2639 (26.8)	966 (41.1)	<0.001
Diuretics, *n* (%)	9385 (77.0)	7720 (78.4)	1665 (70.8)	<0.001
ACEI/ARB drugs, *n* (%)	3624 (29.7)	3345 (34.0)	279 (11.9)	<0.001
SAPS II score	42.1 ± 14.1	40.3 ± 13.4	49.5 ± 14.8	<0.001
SOFA score	3.0 [2.0,5.0]	3.0 [2.0,4.0]	4.0 [2.0,5.0]	<0.001
GCS score	12.0 [7.0,14.0]	13.0 [9.0,14.0]	8.0 [3.0,13.0]	<0.001
Hemoglobin(g/dL)	9.9 ± 2.2	10.0 ± 2.2	9.7 ± 2.3	<0.001
Platelet(K/uL)	178.7 ± 104.7	179.9 ± 101.9	173.5 ± 115.7	0.015
White blood cell(K/uL)	16.2 ± 11.8	16.0 ± 11.1	17.3 ± 14.2	<0.001
Absolute lymphocyte count(K/uL)	1.4 [0.8,2.2]	1.4 [0.8,2.2]	1.2 [0.7,1.9]	<0.001
Anion gap(mEq/L)	16.9 ± 5.1	16.5 ± 4.9	18.8 ± 5.6	<0.001
Bicarbonate(mEq/L)	20.9 ± 5.0	21.2 ± 4.8	19.6 ± 5.6	<0.001
Glucose(mg/dL)	179.5 ± 102.9	176.1 ± 102.2	193.9 ± 104.6	<0.001
Lactate(mmol/L)	3.1 ± 2.7	2.9 ± 2.4	3.8 ± 3.4	<0.001
PO_2_(mm Hg)	82.8 ± 54.3	85.0 ± 53.7	73.6 ± 55.7	<0.001
PCO_2_(mm Hg)	49.3 ± 14.7	49.2 ± 14.2	49.7 ± 16.7	0.207
INR	1.4 [1.2,1.7]	1.3 [1.2,1.6]	1.5 [1.2,2.1]	<0.001
PT(sec)	18.4 ± 12.4	17.7 ± 11.5	21.3 ± 15.4	<0.001
APTT(sec)	33.9 [28.7,47.5]	33.3 [28.5,45.1]	37.8 [29.9,57.9]	<0.001
sodium(mmol/L)	144.8 ± 5.8	144.6 ± 5.4	145.7 ± 7.2	<0.001
potassium(mmol/L)	5.1 ± 0.9	5.1 ± 0.9	5.3 ± 1.0	<0.001
Length of hospital stay (days)	15.4 ± 14.3	16.0 ± 13.7	13.1 ± 16.4	<0.001
Length of ICU stay (days)	5.1 [3.1,9.5]	4.9 [3.1,9.1]	6.1 [3.7,10.9]	<0.001

*Baseline creatinine was defined as the lowest creatinine 48 h before admission.

BMI: body mass index; AKI: acute kidney injury; ARDS: Acute Respiratory Distress Syndrome; SAPS II: Simplified Acute Physiology Score; SOFA: Sequential Organ Failure Assessment; GCS: Glasgow Coma Scale; ACEI/ARB, angiotensin- converting enzyme inhibitor/angiotensin receptor antagonist; PaO_2_: partial pressure of oxygen; PaCO_2_: partial pressure of carbon dioxide; PT: prothrombin time; INR: international normalized ratio; APTT: activated partial thromboplastin time

### Feature selection

Ensemble stepwise feature ranking and selection showed that 11 variables could achieve the best prediction performance (Supplemental Figure 2), and the top 11 variables ranked by feature importance were used as predictors for the prediction model. Ultimately, 11 potential predictors were selected from the original 51 factors. According to the features rank (Supplemental Figure 3), these 11 variables included the GCS score, age, temperature, blood sodium level, absolute lymphocyte count, respiratory rate, SAPS II score, urine output, creatinine level, AKI stage, and body mass index (BMI).

### Model building and evaluation

**Figure 1.**
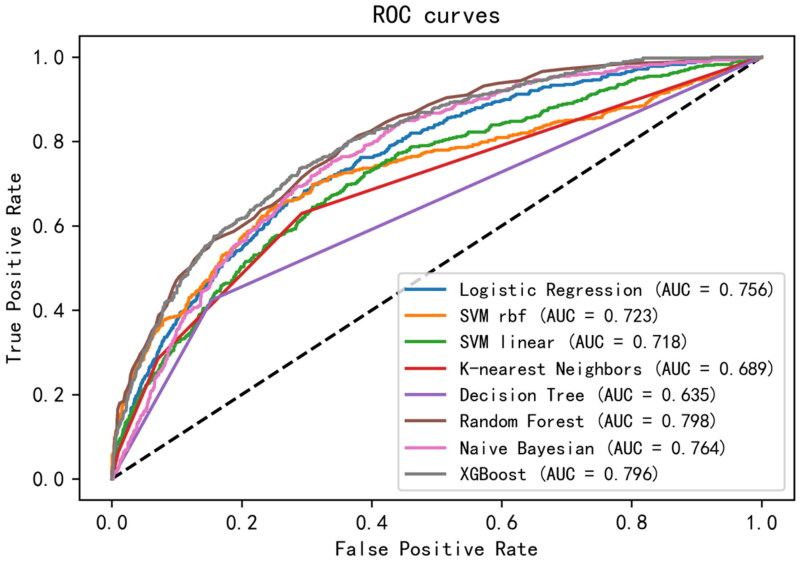
The comparison of ROC curves of the mortality prediction models of seven machine learning algorithms for patients with SA-AKI.

**Figure 2.**
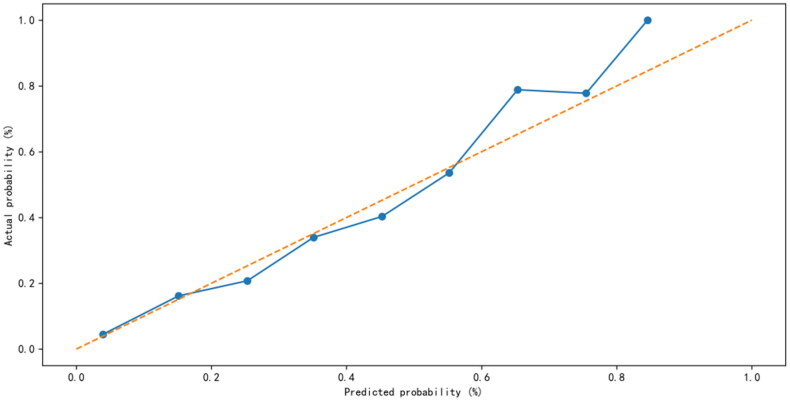
Calibration curve of the RF model for prediction of short-term mortality in patients with SA-AKI.

The 11 selected features were used to establish machine learning prediction models. In the development queues, the RF algorithm has the highest average AUC value for ten-fold cross-validation (0.82, standard deviation: 0.02), with similar ROC-AUC values calculated for each fold (Supplemental Table 1, Supplemental Figure 4). In the test set, the highest AUC value (0.798) was obtained using the RF model, and the lowest AUC value (0.635) was obtained using the decision tree (Figure 1). Table 2 and Figure 2 show that the RF model has great calibration and better differentiation. Compared with Existing severity scores, APACHE II achieves a 0.625 (se = 0.006) C-index score, SOFA achieves a 0.551 (se = 0.007) C-index score.

**Table 2.** The comparison of performance among the prediction models resulted from seven machine learning algorithms that predict the risk of mortality in patients with SA-AKI.

Model	AUC (95%CI)	Accuracy	Precision	F1 scores*	Delong test
Logistic regression	0.756 (0.732–0.779)	0.816	0.569	0.258	<0.001
SVM Linear/rbf	0.718/0.723 (0.691–0.743/0.694– 0.752)	0.809/0.828	1.00/0.671	0.008/0.309	<0.001/<0.001
Naive Bayesian	0.764 (0.741–0.786)	0.795	0.446	0.354	<0.001
XGBoost	0.796 (0.774–0.817)	0.823	0.569	0.414	0.815
Random forest	0.798 (0.774–0.821)	0.832	0.661	0.372	Ref.
K-nearest neighbors	0.689 (0.663–0.714)	0.805	0.487	0.359	<0.001
Decision tree	0.635 (0.611–0.659)	0.765	0.395	0.409	<0.001

*F1 scores= 2* ((precision*recall)/(precision + recall)).

### Explanation of risk factors

**Figure 3.**
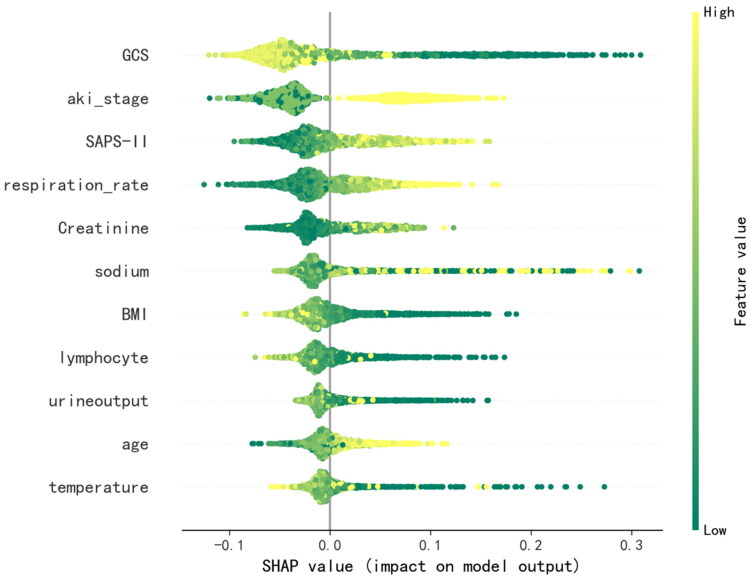
SHAP summary plot of the 11 clinical features of the RF model for prediction of short-term mortality in patients with SA-AKI. GCS: Glasgow Coma Scale score; aki_stage: AKI stage; SAPS-II: Simplified Acute Physiology Score; lymphocyte, absolute lymphocyte count; BMI: body mass index.

The variables, in descending order of SHAP value, contributing to in-hospital mortality risk prediction from most to least important, were GCS score, AKI stage, SAPS II score, respiratory rate, creatinine level, blood sodium level, BMI, absolute lymphocyte count, urine output, age, and temperature (Supplemental Figure 5). Both the SHAP dependence plot (Supplemental Figure 6) and the SHAP summary plot (Figure 3) showed how each baseline variable affected the prognosis of SA-AKI. Each patient’s feature is represented by a single dot that is colored in accordance with an attribution value, with yellow denoting a greater value and Green denoting a lower value, as seen in the SHAP summary figure. Baseline variables with higher SHAP values Increased the probability of dying during hospitalization was higher. Each dot on the SHAP dependence plot represented a patient, showing how the attributed importance of a baseline variable varied as its value increased or decreased. A higher risk of dying during hospitalization was represented by SHAP values greater than zero. According to the SHAP summary plot and SHAP dependence plot, older patients with low GCS score, high AKI stage, reduced urine output, high SAPS II Score, high respiratory rate, low temperature, low absolute lymphocyte count, high creatinine level, dysnatremia, and low BMI were at increased risk of adverse prognostic events during hospitalization.

The SHAP force plot (Supplemental Figure 7) shows profiles of patients who are at high or low risk of developing an outcome during hospitalization in the dataset and demonstrate how a predictive model might aid in the planning of personalized care.

The SHAP force plot visualizes the profiles of patients for outcomes during hospitalization and exemplifies how predictive models can contribute to the planning of personalized care (Supplemental Figure 7). The red section indicates the variables that are at high risk, and giving more attention to these variables may improve the short-term prognosis for that patient.

## Discussion

In this study, we analyzed and effectively screened risk factors associated with mortality during hospitalization in patients with SA-AKI. We used 11 early available clinical parameters to develop and validate a prognostic prediction model for these patients using an RF algorithm, which had better discrimination and calibration and outperformed several machine learning algorithms, such as XGBoost, support vector machine, and traditional LR models. To facilitate the interpretation of the decision-making process of the RF algorithm, we used SHAP to explain the predictions, a SHAP dependence plot to show the relationship between features and their impact on the model measurements, and SHAP force plot to demonstrate how the model specifically personalizes the prediction of the patient’s risk of death.

Most of the previous studies have used traditional regression analysis to screen the variables, which can lead to overfitting as it simultaneously ranks, selects, and does not control the number of variables [13]. Although machine learning methods can deal with a large number of features when constructing models, allowing models containing a large number of features to keep good performance, the models are too complex for clinical utilization. Compare to the Least Absolute Shrinkage and Selection Operator (LASSO) or a single machine learning method, ensemble feature selection can avoid overfitting better and improve the generalizability of trained models. Compared with similar previous studies [29–31], the predictive model developed in this study shows good predictive ability, with more concise predictors and further visualizations. Its visualization and interpretability make the individualized application of the model in the clinic possible.

This study identified GCS score, AKI stage, SAPS II score, respiratory rate, creatinine level, blood sodium level, BMI, absolute lymphocyte count, urine output, age, and temperature as predictors in the development of the RF model. All selected variables were found to be associated with the risk of death in patients with SA-AKI or sepsis in previous studies [31–35]. Immune system dysregulation and release of inflammatory factors are direct pathophysiological mechanisms of kidney injury in SK-AKI. Abnormal lymphocyte counts reflecting cellular immune dysregulation may exacerbate the risk of death. Our study illustrated that lymphocyte count is a risk factor for death, as consistent with previous studies. Previous studies found that persistent lymphocytopenia in sepsis patients is associated with mortality due to sepsis triggering a systemic cytokine-chemokine response, which often leads to lymphocytopenia and may be associated with the inflammatory response and disturbed immune status [36]. Absolute lymphocyte value is one of the characteristics that now receive less attention but will serve as a foundation for future studies. Most of the remaining variables correlate with the patient’s systemic status and the severity of the inflammatory response, especially indicators such as urine output, age, and GCS score, which may be related to the fact that most patients with septic AKI in the ICU die from infectious shock or an exacerbation of the systemic inflammatory response. Some of these variables, such as creatinine level, were correlated with the renal condition, suggesting that the risk of death in patients with sepsis complicated by AKI may be related to the cumulative degree of renal lesions.

In addition, general severity scores are now widely used in critical care units to evaluate patients’ status, such as the SAPS II score and SOFA score. But it does not perform well in terms of mortality prediction in patients with SA-AKI [29]. This is likely because of the complex interactions between immune mechanisms, inflammatory cascade activation, and coagulation pathway disorders in patients with SA-AKI. Moreno-Torres V et al. comodeled red blood cell distribution width (RDW) with SOFA and SAPS II scores, and after adjustments, the model had enhanced predictive efficacy for in-hospital mortality in patients with sepsis [37]. We used the scores as variables in a clinical prediction model and increased the weight of other predictors by adding other variables, both to further improve the original scoring system and to develop new predictive models to assist in clinical decision-making and individualized patient care plan development.

MIMIC-IV is a sizable, publicly accessible critical care database, the data include demographics, vital signs, laboratory results, diagnoses, and hospital discharge information. Its well-established data and meticulous observation records have been extensively used in academic research [32, 38,39]. It is difficult to detect correlations between the data variables in the database when using ordinary regression methods due to their complicated linkages and nonlinear variable interactions, leading to missing important information. The application of machine learning techniques and the fusion of large data have enabled the development of models that are more complicated and perform better than conventional LR methods. In earlier research, machine learning techniques were mainly viewed as ‘black boxes’, offering little insight into how the technology predicts results and how the variables influence the occurrence of the outcomes [40]. The development of Shapley additive explanations adds more actionability by enabling the interpretation and visualization of machine learning technique results.

This study offers a variety of advantages. A prediction model for in-hospital mortality in patients with SA-AKI was developed using machine learning methods with reliable and stable results, and how each variable influences in-hospital death in patients with SA-AKI was analyzed. It can assist in treatment strategies for SA-AKI patients. The risk factors associated with the risk of death during hospitalization in patients with SA-AKI were investigated with a large amount of information from patient data on common clinical parameters.

However, this study also has several limitations. First, MIMIC-IV is a single-center database with rather short-term follow-up. This study may suffer from selection bias and may omit valid information not recorded in the database. Second, the inference process of the machine learning approach still has many unexplained parts, which may limit the generalization and application of the model. Finally, we only examined the parameters of septic AKI patients within 24 h after ICU admission and did not assess dynamic changes. In future studies, the predictive performance of the model for S-AKI needs to be further evaluated through external validation on a larger scale.

## Conclusion

In conclusion, we developed a 11-variable prediction model of RF for predicting the risk of in-hospital mortality in patients with SA-AKI by using a machine learning prediction model based on the MIMIC-IV database. The RF prediction model, which included the GCS score, age, temperature, blood sodium level, absolute lymphocyte count, respiratory rate, SAPS II score, urine output, creatinine level, AKI stage, and BMI, was validated and determined have good discrimination, calibration and clinical practicality in predicting the short-term prognosis of ICU patients with SA-AKI.

## Supplementary Material

Supplemental Material

## Data Availability

The datasets analyzed for this study can be found in the MIMIC-IV (https://mimic.mit.edu/) [11].

## Patient and public involvement

Patients and/or the public were not involved in the design, or conduct, or reporting, or dissemination plans of this research.
