# Supplementary material for: Machine learning-based prediction of in-hospital mortality for critically ill patients with sepsis-associated acute kidney injury
Source: Ren Fail. 2024 Feb 18;46(1):2316267. doi: 10.1080/0886022X.2024.2316267 (PMC10878338; doi:10.1080/0886022X.2024.2316267)
Supplement: Supplemental Material [file IRNF_A_2316267_SM0869.pdf]

Supplemental material

Supplemental Table 1. Comparison of the average ROC-AUC values of seven machine learning algorithms for ten-fold cross-validation in the development queue.

Supplemental Table 2. Comprehensive list of the International Classification of Diseases codes

Supplemental Figure 1: The flow diagram of patient enrolment.

Supplemental Figure 2:Average predictor performance for different numbers of feature sets.

Supplemental Figure 3:Ranking the importance of the top-11 variables in Ensemble Step-Wise Feature Ranking and Selection process.

Supplemental Figure 4:Average ROC-AUC values of seven machine learning algorithms for ten-fold cross-validation in the development queue.

Supplemental Figure 5:Importance matrix plot of SHAP value of 11 predictors derived from the Random Forest (RF) model for prediction mortality in patients with sepsis associated AKI （SA-AKI).

Supplemental Figure 6:SHAP dependence plot of 11 predictors in the RF mortality model for patients with SA-AKI.

Supplemental Figure 7. SHAP force plot for two cases in the dataset at high (A) or low (B) risk of developing an outcome from the RF model for prediction mortality in patients with SA-AKI.

Supplemental Table 1. Comparison of the average ROC-AUC values of seven machine learning algorithms for ten-fold cross-validation in the development queue.

Model	Average AUC	Standard Deviation
Logistic Regression	0.79	0.02
SVM Linear/rbf	0.69/0.76	0.05/0.02
Naive Bayesian	0.78	0.01
XGBoost	0.81	0.01
Random Forest	0.82	0.02
K-nearest Neighbors	0.69	0.03
Decision Tree	0.64	0.02

Supplemental Table 2. Comprehensive list of the International Classification of Diseases codes

Comorbidity	ICD version	ICD code
Hypertension	10	I10 ,I15
ARDS	10	J80
Myocardial infarction	9/10	412 , 410<br>/I21 , I22 , I252
Congestive Heart failure	9/10	428 , 39891 , 40201 , 40211 , 40291 , 40401 , 40403 , 40411 , 40413 , 40491 , 40493 , 4254 , 4255 , 4256 , 4257 , 4258 , 4259<br>/I43 , I50 , I099 , I110 , I130 , I132 , I255 , I420 , I425 , I426 , I427 , I428 , I429 , P290
Cerebrovascular disease	9/10	430 , 431 , 432 , 433 , 434 , 435 , 436 , 437 , 438 , 36234<br>/G45 , G46 , I60 , I61 , I62 , I63 , I64 , I65 , I66 , I67 , I68 , I69 , H340
Chronic pulmonary disease	9/10	490 , 491 , 492 , 493 , 494 , 495 , 496 , 497 , 498 , 499 , 500 , 501 , 502 , 503 , 504 , 505 , 4168 , 4169 , 5064 , 5081 , 5088<br>/J40 , J41 , J42 , J43 , J44 , J45 , J46 , J47 , J60 , J61 , J62 , J63 , J64 , J65 , J66 , J67 , I278 , I279 , J684 , J701 , J703
Diabetes	9/10	2500 , 2501 , 2502 , 2503 , 2508 , 2509 , 2504 , 2505 , 2506 , 2507<br>/E100 , E10l , E106 , E108 , E109 , E110 , E111 , E116 , E118 , E119 , E120 , E121 , E126 , E128 , E129 , E130 , E131 , E136 , E138 , E139 , E140 , E141 , E146 , E148 , E149 , E102 , E103 , E104 , E105 , E107 , E112 , E113 , E114 , E115 , E117 , E122 , E123 , E124 , E125 , E127 , E132 , E133 , E134 , E135 , E137 , E142 , E143 , E144 , E145 , E147
Renal disease	9/10	582 , 585 , 586 , V56,5880 , V420 , V451 ,5830,5831,5832,5833,5834,5835,5836,5837,40301 , 40311 , 40391 , 40402 , 40403 , 40412 , 40413 , 40492 , 40493<br>/N18 , N19,I120 , I131 , N032 , N033 , N034 ,N035 , N036 , N037 , N052 , N053 , N054 , N055 , N056 , N057 , N250 , Z490 , Z491 , Z492 , Z940 , Z992

Comorbidity	ICD version	ICD code
Malignant cancer	9/10	2386 , BETWEEN '140' AND '172', BETWEEN '1740' AND '1958', BETWEEN '200' AND '208' /C43 , C88 ,BETWEEN 'C00' AND 'C26', BETWEEN 'C30' AND 'C34', BETWEEN 'C37' AND 'C41', BETWEEN 'C45' AND 'C58', BETWEEN 'C60' AND 'C76',BETWEEN 'C81' AND 'C85', BETWEEN 'C90' AND 'C97'

Abbreviation: ARDS,Acute Respiratory Distress Syndrome;

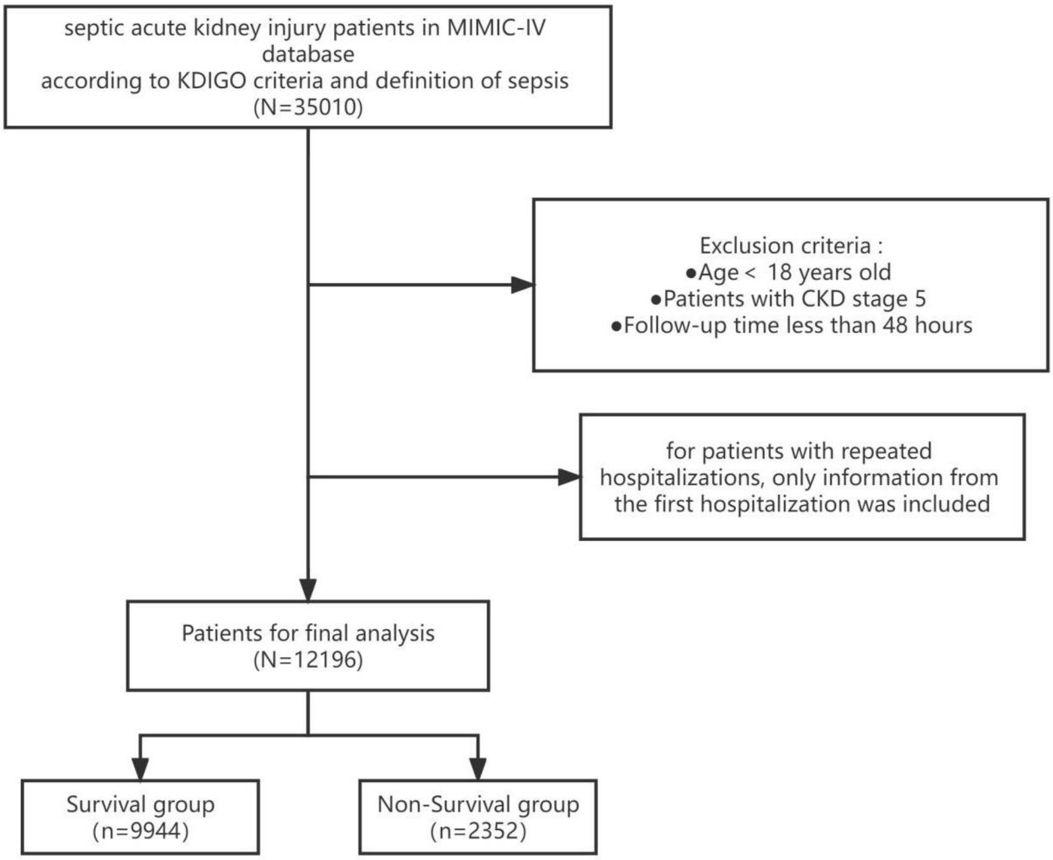

Supplemental Figure 1. The flow diagram of patient enrolment.

Abbreviation: MIMIC, Medical Information Mort for Intensive Care;CKD,chronic kidney disease

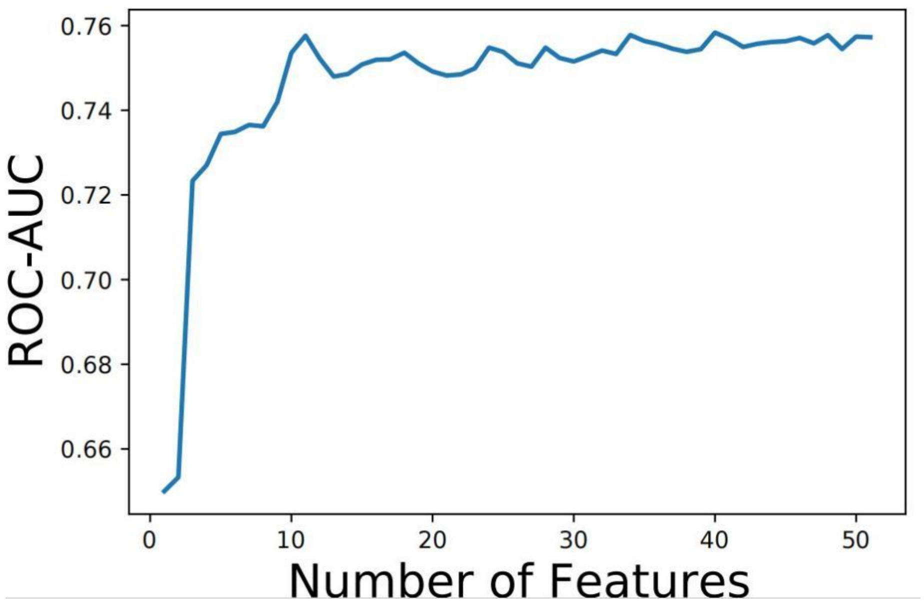

Supplemental Figure 2.   Average predictor performance for different numbers of feature sets.

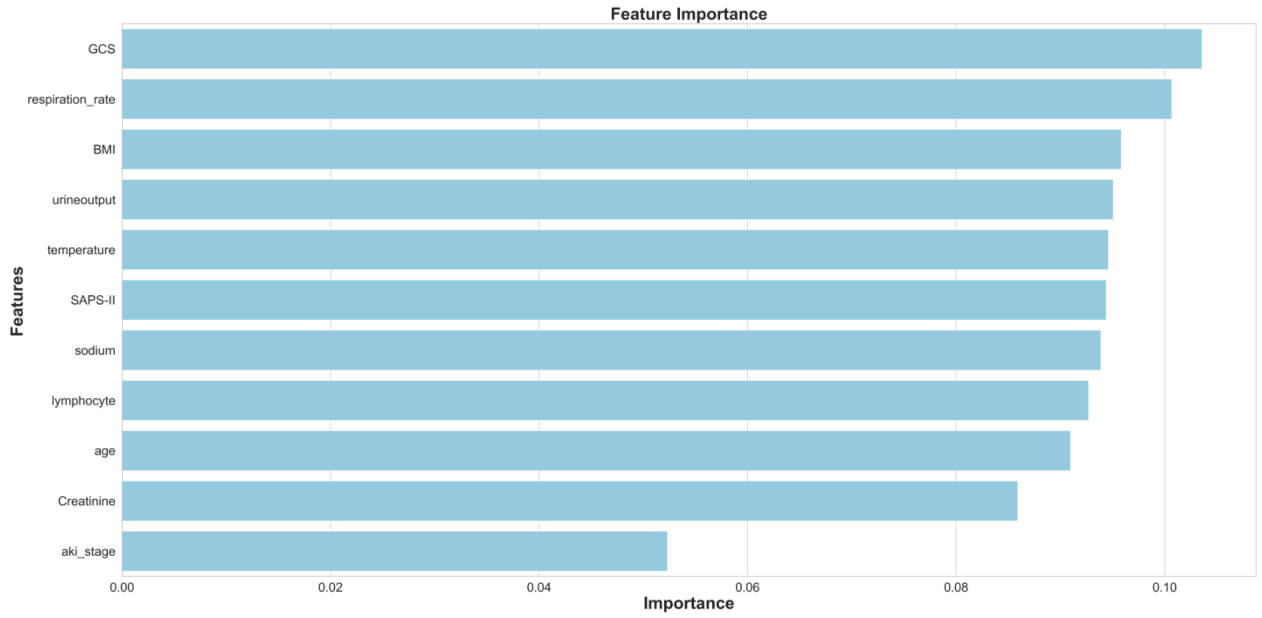

Supplemental Figure 3. Ranking the importance of the top-11 variables in Ensemble Step-Wise Feature Ranking and Selection process.

Abbreviation: GCS,Glasgow Coma Scale score; aki_stage,AKI stage;

SAPS-II,Simplified Acute Physiology Score; lymphocyte,absolute lymphocyte count; BMI, body mass index.

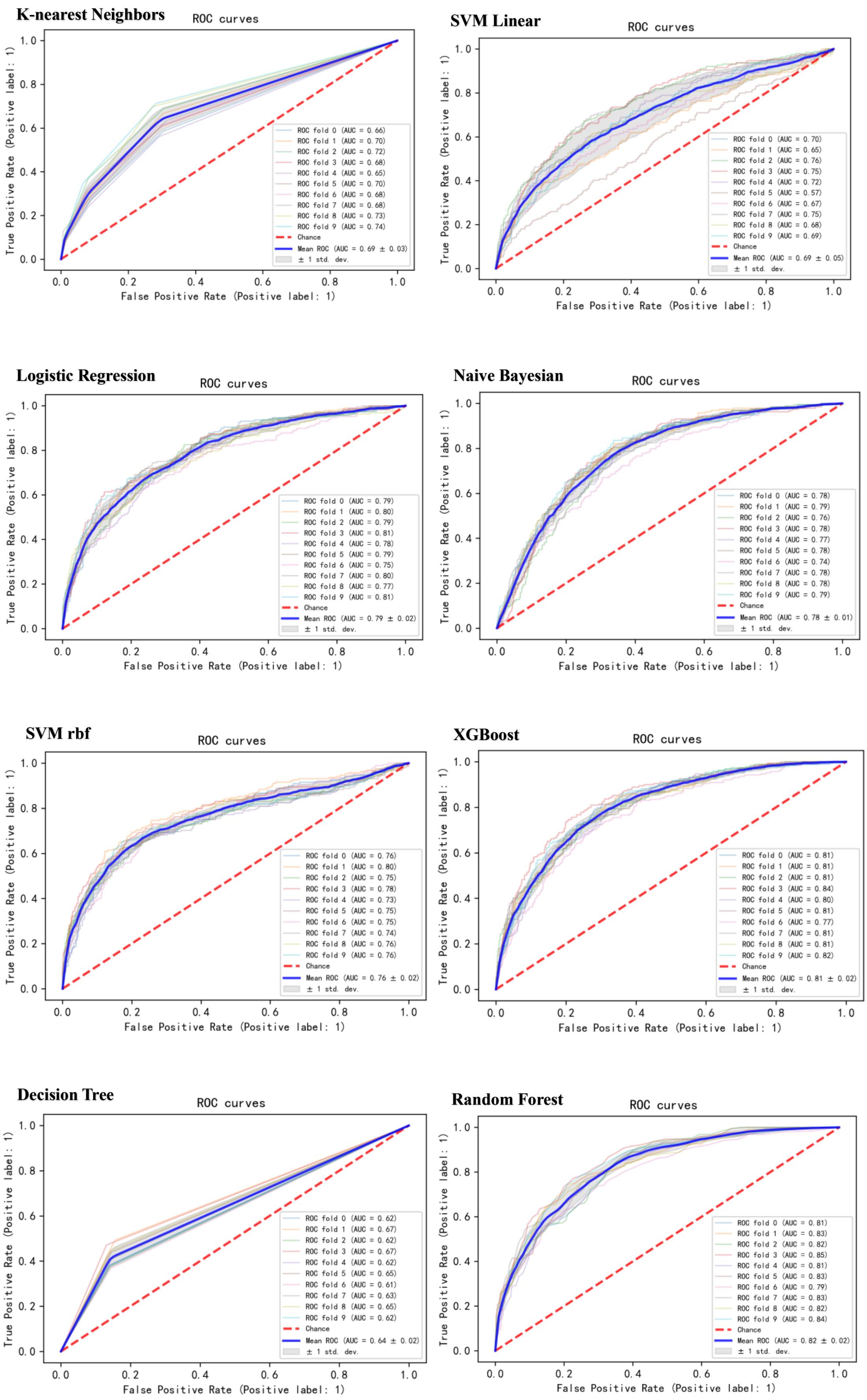

Supplemental Figure 4. Average ROC-AUC values of seven machine learning

algorithms for ten-fold cross-validation in the development queue.

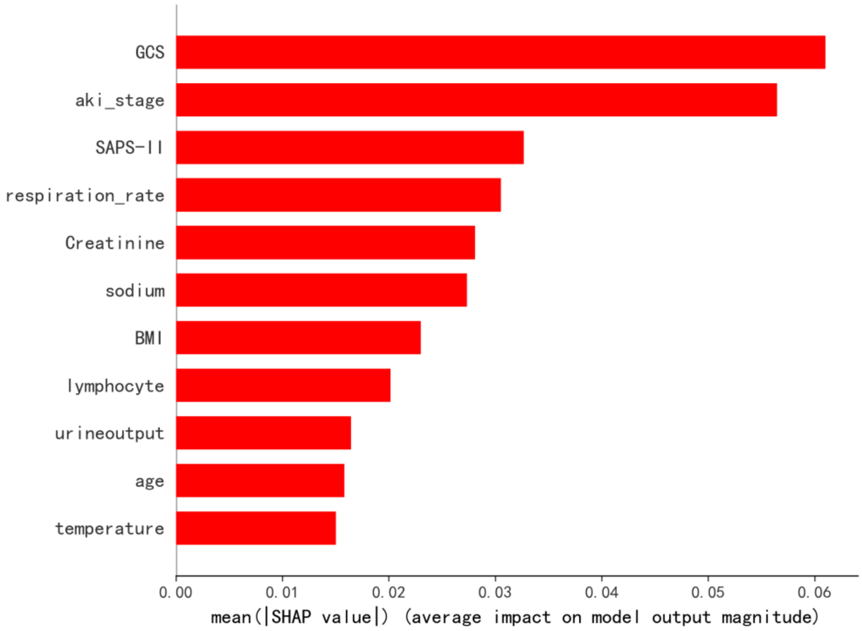

Supplemental Figure 5. Importance matrix plot of SHAP value of 11 predictors derived from the Random Forest (RF) model for prediction mortality in patients with sepsis associated AKI（SA-AKI).

Higher SHAP value means a more important feature.

Abbreviation: GCS,Glasgow Coma Scale score; aki_stage,AKI stage; SAPS-II,Simplified Acute Physiology Score; lymphocyte,absolute lymphocyte count; BMI, body mass index.

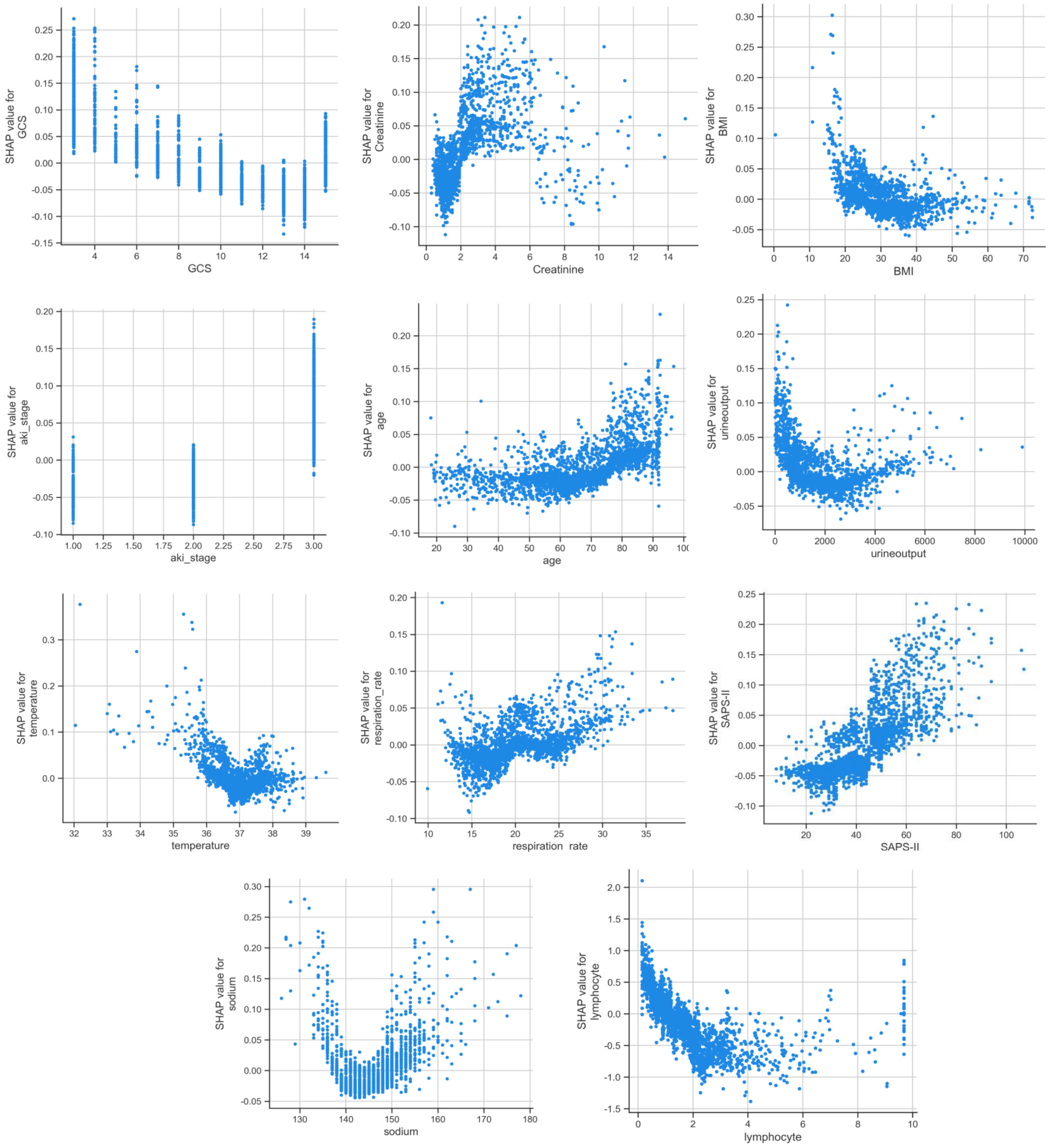

Supplemental Figure 6. SHAP dependence plot of 11 predictors in the RF mortality model for patients with SA-AKI.

The SHAP dependence plot shows how one variable affects the forecast of the RF model.When the value of the variable changes and the SHAP value exceeds 0, it indicates that the risk of in-hospital death of the patient increases under the changing trend of the variable.Abbreviation: Abbreviation: GCS,Glasgow Coma Scale score; aki_stage,AKI stage; SAPS-II,Simplified Acute Physiology Score; lymphocyte,absolute lymphocyte count; BMI, body mass index.

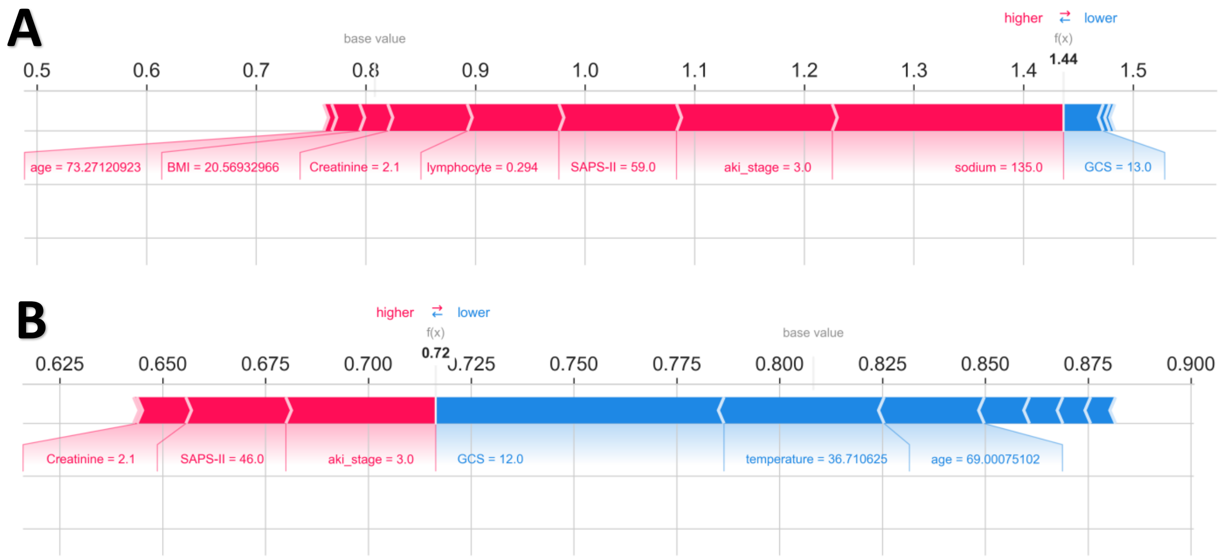

Supplemental Figure 7. SHAP force plot for two cases in the dataset at high (A) or low (B) risk of developing an outcome from the RF model for prediction mortality in patients with SA-AKI.

Red color parts indicate higher risk of mortality than blue color parts for the patient.
